# The Elongation Factor GmEF4 Is Involved in the Response to Drought and Salt Tolerance in Soybean

**DOI:** 10.3390/ijms20123001

**Published:** 2019-06-19

**Authors:** Yuan Gao, Jian Ma, Jia-Cheng Zheng, Jun Chen, Ming Chen, Yong-Bin Zhou, Jin-Dong Fu, Zhao-Shi Xu, You-Zhi Ma

**Affiliations:** 1 Institute of Crop Science, Chinese Academy of Agricultural Sciences (CAAS)/National Key Facility for Crop Gene Resources and Genetic Improvement, Key Laboratory of Biology and Genetic Improvement of Triticeae Crops, Ministry of Agriculture, Beijing 100081, China; gaoyuan9407@126.com (Y.G.); chenjun01@caas.cn (J.C.); chenming02@caas.cn (M.C.); zhouyongbin@caas.cn (Y.-B.Z.); mayouzhi@caas.cn (Y.-Z.M.); 2 College of Agronomy, Jilin Agricultural University, Changchun 130118, China; majian197916@jlau.edu.cn; 3 Anhui Science and Technology University, Fengyang 233100, Anhui, China; zhengjiachengx2016@126.com

**Keywords:** EF1αs, genome-wide analysis, drought tolerance, salt tolerance, hairy root assay, soybean

## Abstract

Growing evidence indicates that elongation factor 1α (EF1α) is involved in responses to various abiotic stresses in several plant species. Soybean EF1α proteins include three structural domains: one GTP-binding domain and two oligonucleotide binding domains that are also called as domain 2 and domain 3. In this study, 10 EF1α genes were identified in the soybean genome. We predicted structures of different domains and analyzed gene locations, gene structures, phylogenetic relationships, various *cis*-elements, and conserved domains of soybean EF1αs. The expression patterns of 10 EF1α genes were analyzed by quantitative real-time PCR (qRT-PCR). Under drought stress, soybean EF1α genes were upregulated in varying degrees. In particular, *GmEF4* was upregulated under drought and salt treatments. Compared to the drought- and salt-treated empty vector (EV)-control plants, drought- and salt-treated *GmEF4*-overexpressing (OE) plants had significantly delayed leaf wilting, longer root, higher biomass, higher proline (Pro) content, and lower H_2_O_2_, O_2_^−^, and malondialdehyde (MDA) contents. Thus, this study provides a foundation for further functional genomics research about this important family under abiotic stress.

## 1. Introduction

Transcript elongation factors (EFs) mediate various growth processes, including the regulation, proliferation, and differentiation of cells [1]. In eukaryotes, EF1 includes α, β, γ, and δ subunits. The α-subunit (EF1α) is considered to be one of the most abundant soluble proteins in cell cytoplasm, where it constitutes up to 3%–10% of the total soluble protein content [2].

It is reported that EF1α plays an essential role in protein biosynthesis and proteolysis. EF1α can carry aminoacyl-tRNAs to the binding sites of ribosomes in protein synthesis. Archaeal EF1α, as a carrier GTPase protein, transfers both aRF1 and aPelota to the ribosome. Archaeal EF1α is associated with aRF1/aPelota, which confirms the absence of specific GTPases for aRF1 and aPelota in archaeal species [3]. In the anabolic metabolism process, protein synthesis was activated by the association between EF1α and AA·tRNA/GTP, but is inactive in proteolysis. By contrast, with the catabolic metabolism process, EF primarily exists as the hydrolyzed form, so EF1α is required for ubiquitin-dependent proteolysis [4].

Several EF1α members were reported to be involved in responses to abiotic stresses in plants. Several reports proposed that the expression of EF1α is related to cold stress in barley and maize [5,6]. Growing evidence indicates that EF1α expression in various plant species changes in response to different stressors, including high and low temperatures, salinity, water deficits, and pollutants [7]. Thus, these results established that EF1α genes may play an important role in the regulation of translation during abiotic stresses.

Soybean is an important economic crop, especially for Chinese. Limited water resources have become one of the main restrictive factors for soybean cultivation and production in China. A previous study has shown that *Glomus mosseae* can improve the drought resistance of soybean [8]. Increasing soybean yield can also be achieved by selecting and cultivating soybean genotypes with higher stem strength [9]. Although several EF1α genes have been well characterized in several plant species, their roles under abiotic stress are still unclear and need further research. In this study, we identified 10 genes of soybean EF1α members and analyzed the chromosome localization, conserved domains, phylogenetic relationships, and expression patterns of *GmEF1αs*. We also conducted an in-depth survey of the expression patterns of *GmEF1αs* in responses to abiotic stress and exogenous hormones. Among them, *GmEF4* was upregulated under drought and salt treatments. Compared with control plants, *GmEF4*-overexpressing plants exhibited drought and salt tolerant phenotypes. Our results provide a foundation for further research on the EF1α family in response to abiotic stresses.

## 2. Results

### 2.1. Identification, Physical Locations, and Conserved Domains of Soybean EF1αs

**Figure 1:**
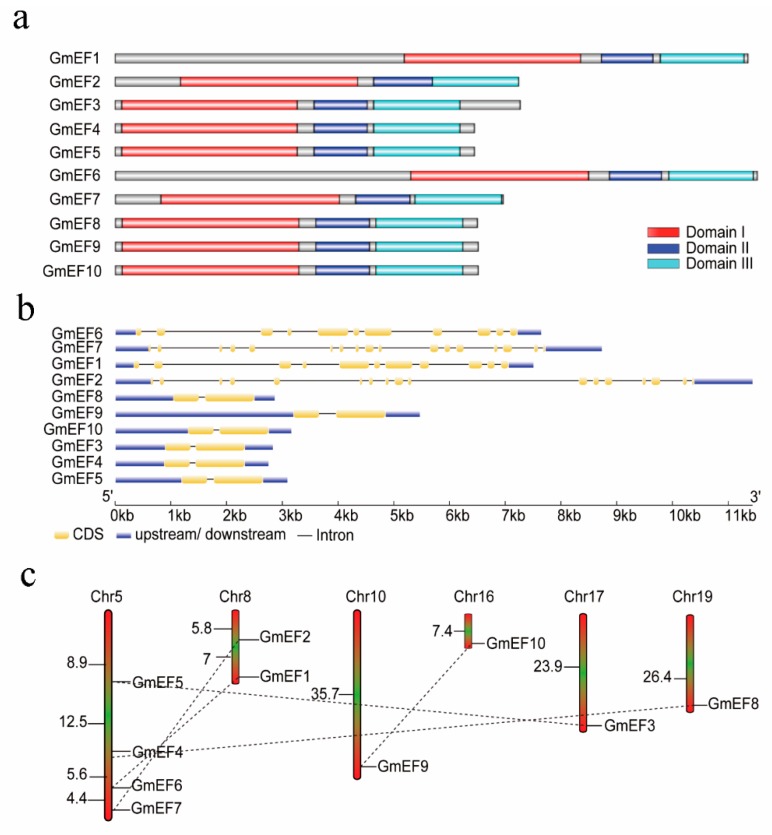
Bioinformatic analysis of GmEF1α proteins and *GmEF1α* genes. (**a**) Structures of EF1α proteins in soybean. (**b**) Intron–exon structures of EF1α genes in soybean. (**c**) Locations and duplications of GmEF1α genes in the soybean genome. Paralogous genes are connected by lines.

The Phytozome database and the SMART program were used as references for the identification of 10 soybean EF1α genes (Table 1). The numbers of amino acids in soybean EF1αs varied widely, ranging from 447 to 792. The predicted isoelectric points (p*I*) of the proteins were diverse, from 4.89 to 9.59 (Table 1). All 10 of the nonredundant GmEF1αs contained three conserved domains: the GTP-binding domain (known as domain I), and two oligonucleotide binding domains (often referred to as domain II and domain III) (TAIR) (Figure 1a). Domain I is important for GDP/GTP binding and GTPase activity. Domain II is responsible for aminoacyl tRNA binding. Domain III is the C-terminal domain.

These genes are distributed on six chromosomes (5, 8, 10, 16, 17, and 19). However, the number of soybean EF1α genes on each chromosome was significantly varied. For example, chromosome 5 and 8 carry four and two EF1α genes, respectively. Each of chromosomes 10, 16, 17, and 19 carries one EF1α gene (Table 1). Using soybean genome repeat area information, five paralogous gene pairs were identified, including GmEF1/6, GmEF2/7, GmEF3/5, GmEF4/8, and GmEF9/10 (Figure 1c).

Nucleotide and deduced amino acid sequences were obtained from Phytozome v9.1 (http://www.phytozome.com/). Gene, CDS, and amino acid identities were determined in this study based on nucleotide or amino acid alignments of the 10 genes using Clustal X 2.0.

### 2.2. The Predicted Structures of Domains in Soybean EF1αs

**Figure 2:**
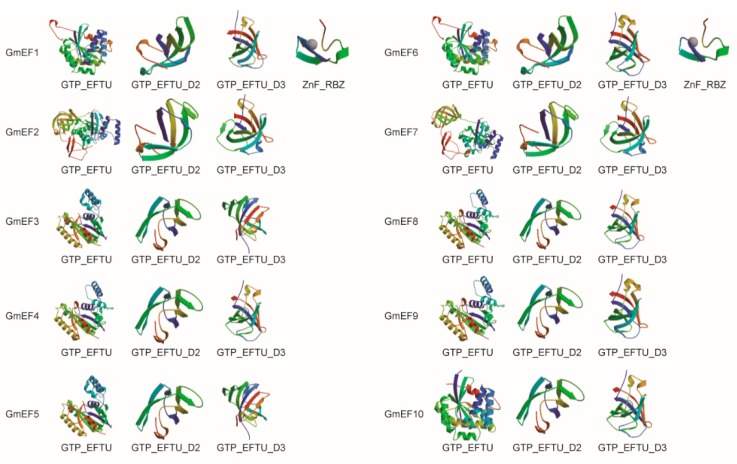
The predicted structures of domains in soybean EF1αs. The predicted structures of soybean EF1αs proteins were analyzed by SMART (http://smart.embl-heidelberg.de/) and were drawn online (https://swissmodel.expasy.org/).

Soybean EF1αs proteins consist of domain 1, domain 2, and domain 3 (Figure 2). All EF-Tu domains include α-helices and β-strands to complex with tRNA and GTP. In particular, GmEF1 and GmEF6 all have the zinc finger domain to make tandem contacts with their target molecule. Domain 2 and domain 3 adopt β-barrel structures, and are involved in binding to charged tRNA and to EF-Tus.

### 2.3. Phylogenetic and Gene Structural Analysis

**Figure 3:**
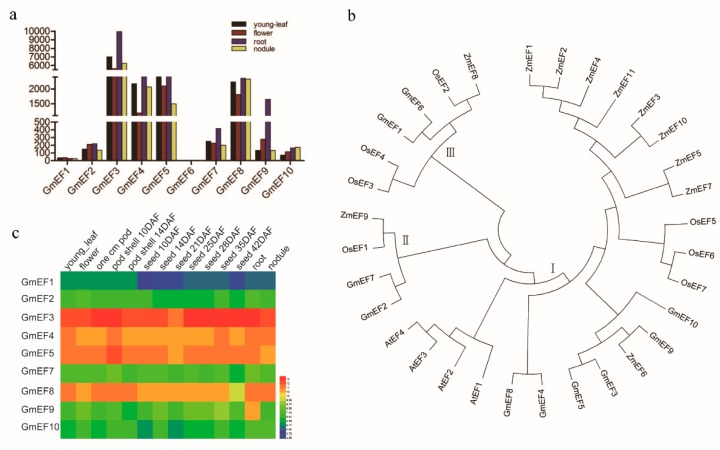
Differential expression analyses of soybean EF1α family members, neighbor-joining phylogenetic tree of EF1α members from *Glycine max* (*Gm*), *Arabidopsis* (*At*), *Zea mays* (*Zm*), and *Oryza sativa* (*Os*), and subcellular localization of GmEF4. (**a**) Expression data were extracted from soybean transcriptome data in SoyBase. In this respect, a transcriptionally active gene is a gene that has two or more sequence reads at one or more of the tested tissues/developmental stages. (**b**) Multiple sequence alignment was performed using the Clustal X program, and the phylogenetic tree was constructed using the neighbor-joining method. *AtEF1* (AT1G07920), *AtEF2* (AT1G07930), *AtEF3* (AT1G07940), *AtEF8* (AT5G60390), *OsEF1* (Os04g20220), *OsEF2* (Os04g58140), *OsEF3* (Os04g50870), *OsEF8* (Os01g02720), *OsEF5* (Os03g08010), *OsEF6* (Os03g08020), *OsEF7* (Os03g08050), *ZmEF1* (GRMZM2G149768), *ZmEF2* (GRMZM2G001327), *ZmEF3* (AC233866), *ZmEF8* (GRMZM2G154218), *ZmEF5* (GRMZM2G343543), *ZmEF6* (GRMZM2G057535), *ZmEF7* (GRMZM2G153541), *ZmEF8* (GRMZM2G084252), *ZmEF9* (GRMZM2G028313), *ZmEF10* (GRMZM2G151193), and *ZmEF11* (GRMZM2G110509). (**c**) An analysis was performed by the Affymetrix soybean gene chip downloaded in SoyBase (https://www.soybase.org/soyseq/), which included the 10 soybean EF1α*s* in the different tissues and development stages. The heatmap was drawn using the software HemI.

To determine the phylogenetic relationships among the soybean EF1αs, a phylogenetic analysis of 10 soybean GmEF1αs, 7 rice OsEF1αs, 11 maize ZmEF1αs, and 4 *Arabidopsis* AtEF1αs was performed by generating a neighbor-joining phylogenetic tree (Figure 3b). The EF1αs were grouped into three clusters. Each cluster is conserved and produces similar proteins. In cluster I, the GmEF8 protein differs from GmEF4 by two amino acids, whereas the GmEF3, GmEF5, GmEF9, and GmEF10 proteins respectively share 96.2%, 96.6%, 97.3%, and 97.5% identity with the GmEF8 protein (Figure 3b and Table 1).

In recent years, research on introns has made significant progress. Studies in mammals, nematodes, insects, fungi, and plants have shown that introns not only regulate gene expression but that they are also involved in gene evolution [10]. Analysis of the gene structure showed that clusters I, II, and III were characterized by 1, 16, and 10 introns, respectively. The intron numbers and intron positions of EF1αs in the same cluster were conserved even though several introns of the same corresponding locations exhibited variable lengths (Figure 1b). This suggests that the intron numbers were established in each cluster before the eudicots and monocots diverged.

### 2.4. Expression Patterns of Soybean EF1αs

To examine the gene expression of the 10 soybean EF1α in different tissues and organs, an expression pattern map was constructed from gene chip data that were downloaded from the soybean genome database and the expression pattern heatmap of the soybean EF1α genes was drawn (Figure 3c and Table S1). The heatmap showed that GmEF3 was highly expressed in all tissues and organs, while GmEF6 was hardly expressed in any tissues and organs and GmEF1 was only weakly expressed in the young leaves, flower, and pod in soybean.

The data analysis of four tissues and organs revealed that all GmEF1α members, except GmEF6, were expressed in the roots, young leaves, nodules, and flowers. Moreover, the expression quantity was similar in the same clusters of EF1α genes. The highest level of EF1α expression was found in cluster I, and included GmEF3, GmEF4, GmEF5, and GmEF8 (Figure 3a). However, GmEF1 in cluster III showed low levels of expression, and no expression was detected for its paralogous gene, GmEF6, perhaps because of weak promoter activity. Unlike other GmEF1αs, the transcription of GmEF9 occurred primarily in root with 5- and 12-fold stronger expression levels in root than in young leaves and flowers, suggesting that the GmEF9 promoter may contain root specific *cis*-elements (Figure 3a).

### 2.5. Soybean EF1αs Contain Various cis-Elements

The analysis of *cis*-elements in the promoters indicated that each soybean EF1α gene, with one exception, carried more than four MYB and MYC elements, which are known to be involved in responses to abiotic stresses [11]. The exception was *GmEF10*, which carried no MYC but had 8 MYB elements. All of these genes contained abscisic acid (ABA)-responsive elements (ABREs), strongly suggesting that these genes may respond to drought and ABA via combination with an ABRE binding protein (AREB) [12]. *GmEF4* contains sixteen MYC elements, which is the highest number observed in the GmEF1α family. In addition, three EF1αs contain dehydration-responsive elements (DREs) (Table 2). The presence of diverse abiotic stress-responsive elements in the promoters of GmEF1αs indicates involvement of these genes in abiotic stress responses. In fact, any abiotic stresses, if sufficiently intense, will result in a non-native conformation (denaturation) of proteins [13]. Thus, members of the GmEF1α family may be required to facilitate the recovery of denatured proteins.

### 2.6. GmEF4 Localized in Cell Membranes

**Figure 4:**
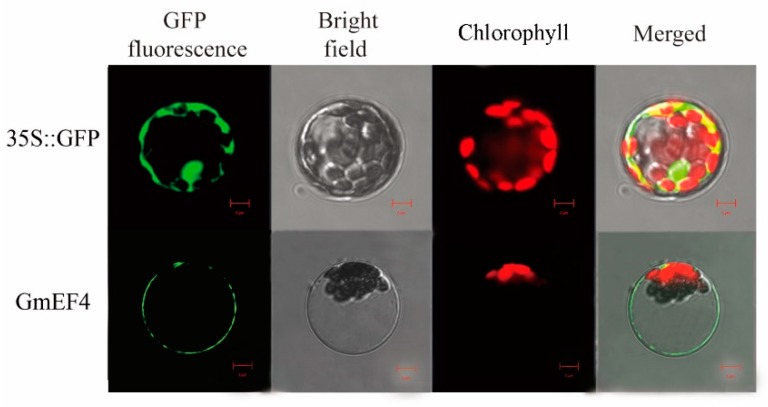
Localization of GmEF4 protein in *Arabidopsis* protoplasts. The construct was transferred into *Arabidopsis* protoplasts and the subcellular localization of the GFP expression was observed in cell membrane. Images were observed under a laser scanning confocal microscope. Bar = 5 μm.

For subcellular localization analysis, the GmEF4 cDNA sequence was fused to the N-terminus of the GFP reporter gene and was inserted into the expression vector under the control of the double CaMV 35S promoter. The construct was transferred into *Arabidopsis* protoplasts and the subcellular localization of GFP expression was observed. We found that the GmEF4-GFP fusion protein was mainly localized in cell membrane, whereas the control GFP was localized uniformly throughout the *Arabidopsis* cells (Figure 4).

### 2.7. Responses of Soybean EF1αs to Drought, Salt, and ABA Stresses

**Figure 5:**
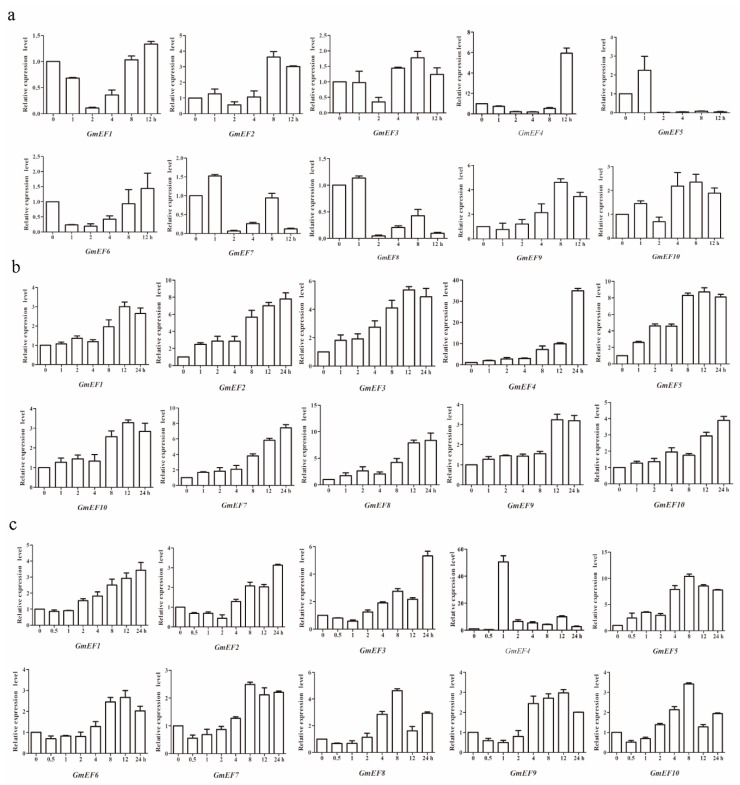
Expression patterns of soybean EF1αs under drought stress, salt stress, and abscisic acid (ABA) treatment. (**a**) Expression levels of the 10 soybean EF1α genes under drought stress, as measured using qRT-PCR. Drought treatment was applied for 0, 1, 2, 4, 8, and 12 h. (**b**) Expression levels of 10 soybean EF1α genes under NaCl treatment for 0, 1, 2, 4, 8, 12, and 24 h. (**c**) Expression levels of the 10 soybean EF1α genes under ABA treatment, as measured using qRT-PCR. ABA treatment was applied for 0, 0.5, 1, 2, 4, 8, 12, and 24 h. The vertical coordinates are fold changes, and the horizontal ordinates are treatment times. The *actin* gene was used as an internal reference. The data are shown as means of three biology repeats ± SD.

To further confirm the drought and salt stress response, the transcription of EF1α genes was investigated by quantitative real-time PCR (qRT-PCR) using soybean seedlings exposed to drought and salt treatments (Figure 5). Based on these results, soybean EF1α genes were expressed differently under drought stress (Figure 5a). Among 10 soybean EF1α members, *GmEF4* reached the highest level under drought treatment at 12 h (>5.5-fold). *GmEF9* was upregulated under drought stress and showed highest levels at 8 h (>4-fold), *GmEF2*, *GmEF10*, and *GmEF3* showed highest levels at 8 h (>3-fold, >2-fold and >1.5-fold). *GmEF1* and *GmEF6* transcriptions were induced by drought and reached a maximum at 12 h (>1-fold). The transcriptions of *GmEF5*, *GmEF7*, and *GmEF8* were induced by drought; the peak at 1 h was followed by a sharp decrease over the next few hours.

In addition, soybean EF1α genes were all upregulated by salt stress (Figure 5b). *GmEF1*, *GmEF3*, *GmEF5*, *GmEF6*, and *GmEF9* transcription were induced by salinity; peaking at 12 h (>2.5-fold, >5-fold, >8-fold, >3-fold, and >3-fold, respectively) was followed by an observed decrease at 24 h. *GmEF2*, *GmEF7*, *GmEF8*, and *GmEF10* transcription were induced by salinity along with time, and increased to their highest level at 24 h (>7-fold, >7-fold, >8-fold, and >3.5-fold, respectively). In particular, *GmEF4* reached the highest level under salt treatment at 24 h (>30-fold).

The transcription of EF1α genes was also investigated by qRT-PCR using soybean seedlings exposed to abscisic acid (ABA) (0, 0.5, 1, 2, 4, 8, 12, and 24 h) treatment (Figure 5). Based on these results, the transcription of all of soybean EF1α genes were induced by ABA and were increased to different degrees (Figure 5c). The expressions of *GmEF5*, *GmEF7*, *GmEF8*, and *GmEF10* reached the highest levels at 8 h (>10-fold, >2-fold, >4-fold, and >3-fold, respectively), while the transcript levels of *GmEF1*, *GmEF2*, and *GmEF3* reached their maximums at 24 h (>3-fold, >3-fold, and >5-fold, respectively). The transcription of *GmEF6* and *GmEF9* reached a maximum at 12 h (both >2.5-fold). Among 10 soybean EF1α members, *GmEF4* reached the highest level under ABA treatment at 1 h (>50-fold). Hence, it may be play important role in the response to drought, salt, and ABA stress.

### 2.8. Overexpression of GmEF4 Significantly Improved Drought Tolerance in Soybean Hairy Roots

**Figure 6:**
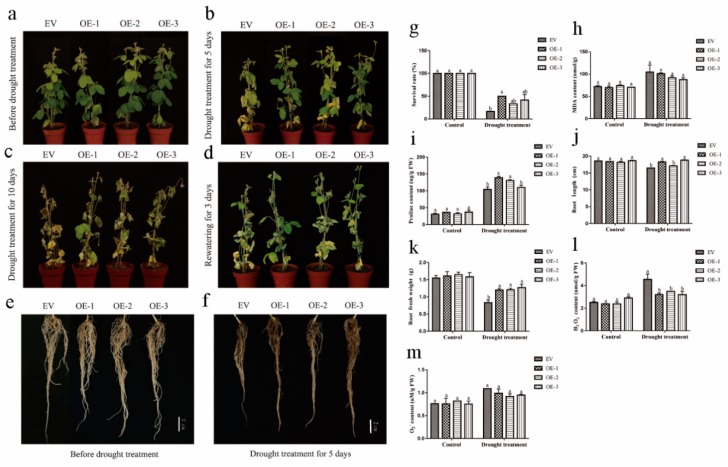
Phenotypic analysis of *GmEF4*-overexpressing (*GmEF4*-OE) and empty vector (EV)-control plants under drought treatment. (**a**) and (**b**,**c**) Phenotypes of *GmEF4*-OE and EV-control transgenic soybean plants under normal and drought conditions. (**d**) Phenotypes of *GmEF4*-OE and EV-control transgenic soybean plants under rewatering conditions. (**e**) and (**f**) Phenotypes of *GmEF4*-OE and EV-control hairy roots under normal and drought conditions. (**g**) Survival of normal and drought-stressed plants. (**h**) Malondialdehyde (MDA) content and (**i**) proline content of *GmEF4*-OE and EV-control plants grown with drought treatment. (**j**) the root length, (**k**) total fresh weight, (**l**) H_2_O_2_ content, and (**m**) O_2_^−^ content of *GmEF4*-OE and EV-control soybean hairy roots grown with drought treatment. The different letters in the bar graphs indicate significant differences at *p* < 0.05 between *GmEF4*-OE and EV-control transgenic soybean.

To further investigate the biological function of GmEF4 in drought response, we used *Agrobacterium rhizogenes*-mediated transformation of soybean hairy roots to generate soybean hairy roots overexpressing *GmEF4* (*GmEF4*-OE). qRT-PCR analysis showed that, compared to the empty vector (EV)-transformed control hairy roots, the roots with the overexpression construct had significantly increased GmEF4 expression (Figure S1). We found that, whereas no significant differences were observed between the EV-control and the *GmEF4*-OE plants under normal growth conditions (Figure 6a), drought treatment caused obvious differences in the growth and physiology of the EV-control and the *GmEF4*-OE plants (Figure 6b,c). Compared with the EV-control, *GmEF4*-OE plants appeared wilted leaf under drought treatment for 5 days. The water loss of EV-control and *GmEF4*-OE plants are severe under drought treatment for 10 days. In addition, the leaves of *GmEF4*-OE plants recover phenotype compared to the EV-control plants after rewatering for 3 days (Figure 6d). We found that whereas no significant differences were observed between the EV-control and the *GmEF4*-OE roots under normal growth conditions (Figure 6e), but compared to the drought-treated EV-control roots, drought-treated *GmEF4*-OE roots were longer (Figure 6f). Drought stress can lead to the accumulation of peroxides. By measuring the contents of H_2_O_2_ and O_2_^−^, we found that the drought-treated *GmEF4*-OE roots have lower H_2_O_2_ content and lower O_2_^−^ content (Figure 6l,m). Drought stress can also lead to the accumulation of proline. By measuring the content of Pro, we found that the content of Pro in the *GmEF4*-OE was significantly higher than that of the EV-control under drought treatment (Figure 6i). The drought-treated *GmEF4*-OE plants have higher survival rate and lower MDA content (Figure 6g,h). The drought-treated *GmEF4*-OE roots have higher biomass and were longer (Figure 6j,k).

### 2.9. Overexpression of GmEF4 Significantly Improved Salt Tolerance in Soybean Hairy Roots

**Figure 7:**
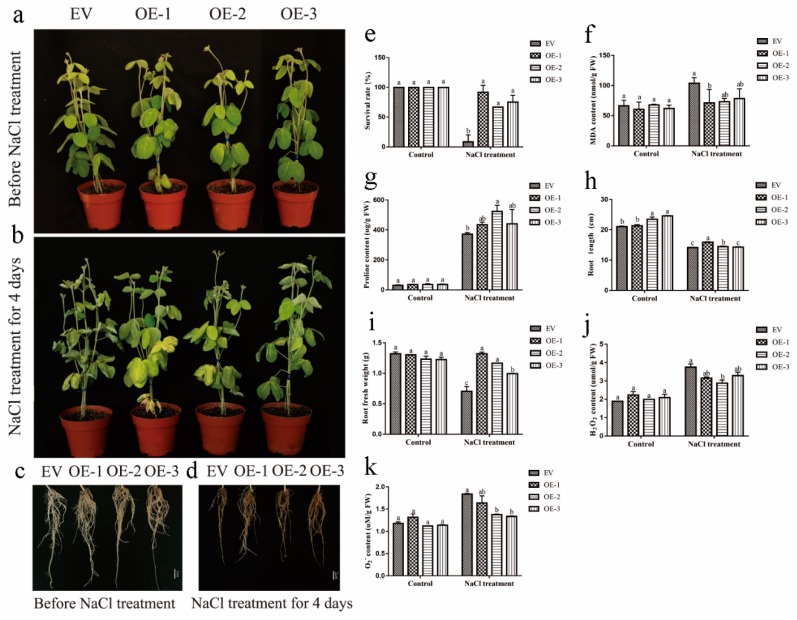
Phenotypic analysis of *GmEF4*-overexpressing (*GmEF4*-OE) and EV-control plants under salt treatment. (**a**) and (**b**) Phenotypes of *GmEF4*-OE and EV-control transgenic soybean plants under normal and salt treatment. (**c**) and (**d**) Phenotypes of *GmEF4*-OE and EV-control hairy roots under normal and salt treatment. (**e**) Survival of normal and salt-stressed plants. (**f**) MDA content and (**g**) proline content of *GmEF4*-OE and EV-control plants grown with salt treatment. (**h**) the root length, (**i**) total fresh weight, (**j**) H_2_O_2_ content, and (**k**) O_2_^−^ content of *GmEF4*-OE and EV-control soybean hairy roots grown with salt treatment. The different letters in the bar graphs indicate significant differences at *p* < 0.05 between *GmEF4*-OE and EV-control transgenic soybean.

To further investigate the roles of GmEF4 in salt response, we used the same methods to generate *GmEF4*-OE plants. No significant differences were observed between the EV-control and the *GmEF4*-OE plants under normal growth conditions (Figure 7a). However, obvious differences in the growth and physiology of the EV-control and the *GmEF4*-OE plants appeared under salt treatment for 4 days (Figure 7b). The *GmEF4*-OE plants have higher biomass than EV-control plants and with fewer wilting of leaves than EV-control plants. Specifically, compared to the EV-control plants, salt-treated *GmEF4*-OE plants have longer roots (Figure 7c,d). The salt-treated *GmEF4*-OE plants have a higher survival rate, lower MDA content, and higher proline content (Figure 7e–g). The salt-treated *GmEF4*-OE roots have higher biomass and longer roots (Figure 7h,i). Salt stress can lead to the accumulation of peroxides. By measuring the contents of H_2_O_2_ and O_2_^−^, we found that the salt-treated *GmEF4*-OE roots have lower H_2_O_2_ content and lower O_2_^−^ content (Figure 7j,k).

### 2.10. Overexpression of GmEF4 Reduced the Content of H_2_O_2_ under Drought and Salt Stresses

**Figure 8:**
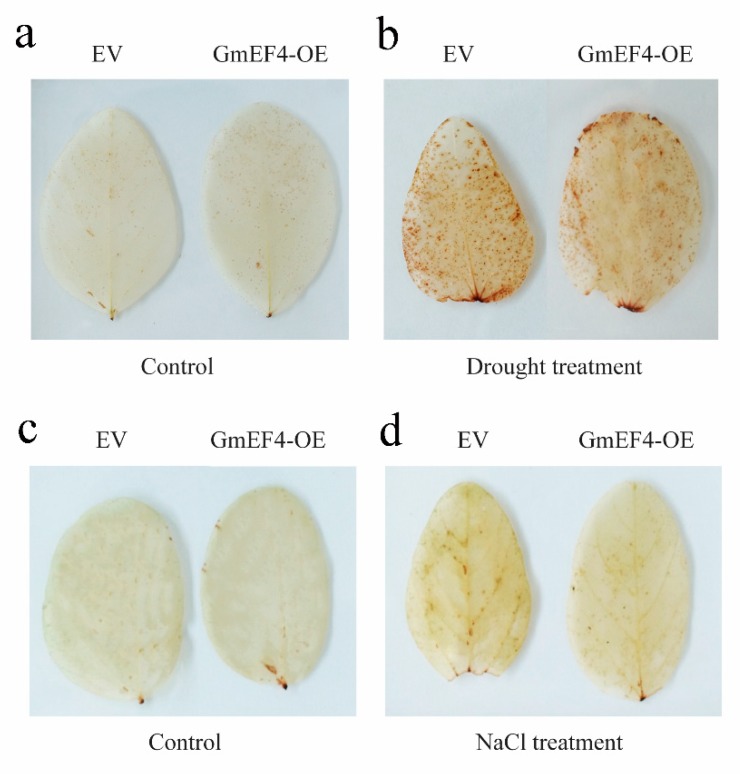
DAB staining of *GmEF4*-overexpressing (*GmEF4*-OE) and EV-control plants under drought and salt treatments. (**a**) and (**b**) DAB staining of *GmEF4*-OE and EV-control transgenic soybean plants under normal and drought treatment. (**c**) and (**d**) DAB staining of *GmEF4*-OE and EV-control transgenic soybean plants under normal and salt treatment.

We used a 3, 3-diaminobenzidine (DAB) solution to detect the H_2_O_2_ content in EV-control and *GmEF4*-OE leaves. No significant difference was observed between the EV-control and *GmEF4*-OE leaves under normal growth conditions (Figure 8a,c), however, the color depth of the *GmEF4*-OE leaves was lower than EV-control leaves under drought and salt treatments (Figure 8b,d). This indicates that the overexpression of *GmEF4* improved drought and salt tolerance in soybean.

## 3. Discussion

During evolution, plants have developed various mechanisms to respond to environmental factors [14,15]. A previous study has shown that higher expression of *GmTIP2;3* can not only enhance the tolerance of plants to osmotic stress, but also effectively improve the tolerance of yeast to drought stress [16]. There are also several reports in barley and maize on the induction of EF1α under other abiotic stresses. In this study, 10 soybean EF1αs were identified and characterized after scanning the current version of the soybean genome. The dicotyledons soybean and *Arabidopsis* contain 10 and 4 EF1α genes, respectively, suggesting the possibility of a past double duplication event in the soybean genome [17] compared to a single duplication in *Arabidopsis* [18].

According to the qRT-PCR analysis, GmEF4 was highly expressed under exogenous ABA stress. It was reported that ABA can improve plant resistance to drought to ensure crops grow normally in challenging environments. We found that the EV-control and *GmEF4*-OE have no significant differences under normal growth conditions, but that drought and salt treatments caused obvious differences in the growth and physiology of the EV-control and the GmEF4 transgenic plants. Specifically, compared to the drought- and salt-treated EV-control plants, drought- and salt-treated *GmEF4*-OE plants had significantly delayed leaf wilting, longer roots, higher biomass, higher proline content, and lower MDA content. Hence, GmEF4 may be involved in the crosstalk of various stresses in plants.

EF1αs are associated with a number of proteins that exert their functional roles in plant biology. In previous study, the plant EF1α has been shown to physically interact with other proteins, suggesting that they may operate together in cellular processes in three ways: via enhanced translation, an interaction with the cytoskeleton, and catalysis phosphorylation in plants. EF1α is directly bound by plant calmodulins (CAMs), which inhibit its ability to bundle microtubules in vitro [19]. Wheat germ EF1α and GTP can bind with TYMV Val-RNA and form a stable complex [20], resulting in the inhibition of minus strand synthesis [21]. EF1α can bind to the PH domain of PLC-γ1. EF1α is regarded as a phosphatidylinositol 4-kinase activator through its interaction with the PH domain [22]. Thus, it is regarded as a multifunctional protein.

EF1α is involved in other cellular process through its interactions with other proteins. For example, ZmEF1α can interact with actin filaments under certain pH conditions (6.0–8.0) to regulate growth processes in plant cells [23]. Recently, it was reported that EF1αs mediate abiotic stress responses. The accumulation of EF1α was induced in ripe wheat under heat stress, which confirmed that EF1α was essential for the response to heat stress in crop [24]. In this study, soybean EF1α members highly respond to drought, salt, exogenous ABA. This could contribute to improving plant resistance to stress conditions through interactions with regulatory factors. This provides a basis for exploring the molecular mechanisms by which the EF1α family plays a specific role.

## 4. Materials and Methods

### 4.1. Genomic Location of Soybean EF1α Loci

To gather probable GmEF1α amino acid sequences candidates, the EF1α-type GTP-binding domain (Pfam: PF00009) was submitted as a query in BLASTP searches of the JGI Glyma1.0 genome version (http://www.phytozome.net/index.php) [25]. A total of 10 *GmEF1α* were identified after filtering out repeated sequences using SMART (http://smart.embl-heidelberg.de/). All nonredundant EF1αs were mapped onto six soybean chromosomes on the basis of information about their corresponding locations in the soybean database (http://soybase.org/).

### 4.2. Analysis of Gene Structure and cis-Acting Elements

An exon–intron structure map was created using GSDS online tools (http://gsds.cbi.pku.edu.cn/) to analyze gene structures and Promoter 2.0 (http://www.cbs.dtu.dk/services/Promoter/) was used and to predict the promoter sequences of the *GmEF1α* genes. Potential *cis*-elements were analyzed using the plant *cis*-element database, PLACE26.0 (http://www.dna.affrc.go.jp/PLACE/signalscan. html).

### 4.3. Genomic and Phylogenetic Relationships and Expression Patterns

Both the EF1α gene sequences and protein sequences of *Arabidopsis*, rice, and maize were acquired from TAIR (http://arabidopsis.org), RGAP (http://rice.plantbiology.msu.edu), and TIGR (http://maize.jcvi.org/), respectively. Clustal X 2.0 was applied in a protein sequence comparison analysis of EF1α*s* in *Arabidopsis*, rice, maize, and soybean. A phylogenetic tree was constructed using the neighbor-joining method in MEGA 5.0 (http://megasoftware.net) using a bootstrap value of 500.

An analysis was performed by the Affymetrix soybean gene chip downloaded in SoyBase (https://www.soybase.org/soyseq/), which included the 10 soybean EF1α*s* in the different tissues and development stages. The heatmap was drawn using the software HemI.

### 4.4. RNA Extraction and qRT-PCR Assays

Isolation of the total RNA from soybean plants under drought and salt stress was performed using an RNA extraction kit (Takara, Shiga, Japan) according to the manufacturer’s recommendations. cDNA synthesis was conducted as previously described [14]. The qRT-PCR expression was measured using an ABI Prism 7500 sequence detection system (Applied Biosystems, Foster City, CA, USA), as described by Liu et al. [26]. Soybean *actin* (U60506) was used as internal controls for the normalization of the template cDNA. All primers used in the study are listed in Table S2.

### 4.5. Agrobacterium rhizogenes-Mediated Transformation of Soybean Hairy Roots

To generate *GmEF4*-OE soybean hairy roots, the coding sequences of *GmEF4* were inserted into the plant transformation vector pCAMBIA3301 driven by the *CaMV* 35S promoter. The resultant constructs were introduced into *Agrobacterium rhizogenes* strain K599, and the bacterium was used to infect soybean hypocotyls by injection, as previously described [27,28]. The infected plants were transferred to a greenhouse and kept at high humidity until hairy roots were generated at the infection site. The original main roots were removed by cutting from 1 cm below the infection site. Seedlings were transplanted into a sieve-like plate containing mixed soil (1:1 vermiculite/humus) and then cultured normally in the greenhouse for 7 days (25 °C, 16 h light/8 h dark photoperiod).

### 4.6. Drought and Salt Tolerance Analyses

For soybean, *GmEF4*-OE and EV-control soybean seedlings with 3~5 cm hairy roots were planted in pots containing mixed soil (1:1 vermiculite/humus) and cultured normally in the greenhouse for 6~8 days, then EV-control and *GmEF4*-OE plants were grown without watering for 10 days. After drought treatment, we rewatered EV-control and *GmEF4*-OE plants for 3 days. For the salt stress assay, EV-control and *GmEF4*-OE plants were treated with 250 mM/L NaCl solution for 4 days [27,29].

### 4.7. Subcellular Localization

Full-length GmEF4 was fused to the N-terminus of the GFP gene under control of the CaMV 35S promoter [14]. Transient expressions of the 35S::GmEF4-GFP fusion construct and the GFP control vector were estimated by introducing the resultant DNAs into *Arabidopsis* protoplasts using a protocol [30]. FM4-64 dye (Molecular Probes, Eugene, OR, USA) was excited at 543 nm, and its fluorescence was recorded using a 650 nm long-pass filter. Subcellular localization of GFP expression in *Arabidopsis* protoplasts was monitored using confocal microscopy after 16 h/22 °C dark incubation using PEG-mediated transformation, as described elsewhere [26]. All transient expression experiments were repeated three times.

### 4.8. Measurements of Fresh Weight and Main Length

The root lengths were evaluated using an Epson Expression 11000XL root system scanning analyzer (Epson, Nagano, Japan). The fresh weights were measured using a Sartorius BSA224S-CW 1/10,000 analytical balance (Sartorius, Beijing, China).

### 4.9. Measurements of MDA Content, Proline Content, H_2_O_2_ Content, and O_2_^−^ Content

For physiological parameter measurements, the leaves of drought- and salt-treated EV-control plants and *GmEF4*-OE plants were harvested for the measurements of MDA content and proline content. The *GmEF4*-OE and EV-control soybean seedlings with 3~5 cm hairy roots were planted in pots and grown in the greenhouse for 6~8 days, and were then grown without watering for 5 days and treated with 250 mM/L NaCl solution for 4 days before being harvested for measurement of H_2_O_2_ content and O_2_^−^ content. The MDA content was assayed according to the protocol at http://www.cominbio.com/uploads/soft/190325/2-1Z325154356.pdf. About 0.1 g of soybean leaf was used and absorbance values at 532 and 600 nm. The MDA content was calculated using the following formula: MDA content (nmol/g FW) = 51.6 × (OD_532_−OD_600_)/0.1. The proline content was assayed according to the protocol at http://www.cominbio.com/uploads/soft/180727/1-1PHGF414.pdf. About 0.1 g of soybean leaf was used and absorbance values at 520 nm. The proline content was calculated using the following formula: Pro content (µg/g FW) = 38.4 × (OD_520_ + 0.0021)/0.1. The H_2_O_2_ content was assayed as previously described (http://www.cominbio.com/uploads/soft/180727/1-1PHGF628.pdf) [31]. About 0.1 g of soybean hairy roots was used and absorbance values at 415 nm. The H_2_O_2_ content was calculated using the following formula: H_2_O_2_ content (µmol/g FW) = 2.67 × (ΔA−0.0006)/0.1. The O_2_^−^ content was assayed as previously described [31] (http://www.cominbio.com/uploads/soft/190326/2-1Z326145U1.pdf). Briefly, 1 mL extract solution was added to 0.1 g soybean hairy roots, ground into tissue homogenate on ice, centrifuged at 4 °C, 10,000 *g* for 20 min, and then the supernatant fraction was collected for reaction according to the manufacturer’s instruction. The O_2_^−^ content was calculated using the following formula: O_2_^−^ content (µmol/g FW) = 148.76 × (ΔA + 0.0027) × 1000/W. Absorbance values were measured with a Varioskan LUX Multimode Microplate Reader (ThermoFisher Scientific, Waltham, MA, USA). Each experiment was repeated three times.

### 4.10. DAB Staining

The *GmEF4*-OE and EV-control soybean seedlings with 3~5 cm hairy roots were planted in pots and grown in the greenhouse for 6~8 days, then were grown with and without watering for 5 days and treated with and without 250 mM/L NaCl solution for 4 days before being harvested for the DAB staining assay. The detached leaves were immersed into DAB solution (Solarbio, Beijing, China) for 14 h and transferred to 75% ethanol for decoloration until the samples become white [29].

## 5. Conclusions

We predicted the structures of different domains and analyzed gene locations, gene structures, phylogenetic relationships, various *cis*-elements, and conserved domains of soybean EF1αs. The expression patterns of 10 EF1α genes were analyzed by quantitative real-time PCR (qRT-PCR). We found that *GmEF4* was upregulated under drought, salt, and ABA treatments. Compared with the EV-control plants, *GmEF4*-OE plants exhibited drought and salt tolerant phenotypes. Thus, it provides a foundation basis for further functional genomics research about this important family under abiotic stress.

## Figures and Tables

**Table 1:** Details of the 10 soybean elongation factor 1α (EF1α) proteins, including sequence ID, protein sequence length, predicted molecular weight (MW), predicted isoelectric point (p*I*), chromosomal location, and predicted functional domains.

Gene	Gene ID	Amino Acids	MW (Da)	p*I*	Chromosome	Domain I	Domain II	Domain III
*GmEF1*	Glyma08g12790	787	85,982.9	6.43	8	360–579	605–669	678–782
*GmEF2*	Glyma08g05570	504	56,282.4	4.89	8	82–302	322–389	395–502
*GmEF3*	Glyma17g23900	447	49,387.0	9.56	17	9–227	248–314	322–429
*GmEF4*	Glyma05g24110	447	49,254.9	9.58	5	9–227	248–314	322–429
*GmEF5*	Glyma05g11630	447	49,347.0	9.56	5	9–227	248–314	322–429
*GmEF6*	Glyma05g29675	792	86,366.4	6.9	5	365–584	610–674	683–787
*GmEF7*	Glyma05g34120	479	53,359.7	5.16	5	57–277	297–364	370–477
*GmEF8*	Glyma19g07240	447	49,270.9	9.58	19	9–227	248–314	322–429
*GmEF9*	Glyma10g35700	448	49,491.1	9.59	10	9–227	248–314	322–429
*GmEF10*	Glyma16g07350	447	49,397.1	9.56	16	9–227	248–314	322–429

**Table 2:** Distribution and numbers of *cis*-acting elements in soybean EF1α genes.

Genes	*GmEF1*	*GmEF2*	*GmEF3*	*GmEF4*	*GmEF5*	*GmEF6*	*GmEF7*	*GmEF8*	*GmEF9*	*GmEF10*
ABRE	1	1	7	6	6	1	1	8	6	1
DRE	0	1	0	0	1	0	0	0	1	0
LTRE	1	1	0	1	0	0	0	2	1	0
MYB	13	11	16	7	9	5	16	10	13	8
MYC	16	11	10	16	12	4	12	10	10	0

## Supplementary Materials

Supplementary materials can be found at https://www.mdpi.com/1422-0067/20/12/3001/s1. Table S1; Analysis data of 10 GmEF1αs genes for the heat map. Table S2; Primers used in the paper. Figure S1; qRT-PCR analyses of GmEF4 expression in *GmEF4*-OE and EV-control transgenic soybean hairy roots.

## Abbreviations

EF1α	elongation factor 1α
p*I*	isoelectric points
ABREs	ABA-responsive elements
DREs	dehydration-responsive elements
EF-Tu	elongation factor thermo unstable
ABA	abscisic acid
qRT-PCR	quantitative real-time PCR
CAMs	calmodulins
GFP	green fluorescent protein
MDA	malondialdehyde
Pro	proline
DAF	days after flowering
