# Supplementary material for: The Elongation Factor GmEF4 Is Involved in the Response to Drought and Salt Tolerance in Soybean
Source: Int J Mol Sci. 2019 Jun 19;20(12):3001. doi: 10.3390/ijms20123001 (PMC6627591; doi:10.3390/ijms20123001)
Supplement: Supplementary file 1 [file ijms-20-03001-s001.pdf]

Table S1: Analysis datas of ten GmEF1αs genes for heat map.

	young_leaf	flower	one cm pod	pod shell 10DAF	pod shell 14DAF	seed 10DAF	seed 14DAF	seed 21DAF	seed 25DAF	seed 28DAF	seed 35DAF	seed 42DAF	root	nodule
GmEF1	36	41	36	39	37	20	13	12	23	26	32	8	28	27
GmEF2	151	211	121	152	101	107	94	64	95	74	118	59	220	137
GmEF3	7018	5629	8923	12365	7639	5485	7294	3076	9721	9849	14543	8396	9944	6251
GmEF4	2173	1186	1717	2727	1831	1180	1932	1174	1891	1849	2156	1017	2512	2066
GmEF5	3210	2101	3479	4499	2467	2384	2849	1293	2635	2545	3982	2180	3319	1494
GmEF7	253	229	201	284	137	166	195	113	230	130	245	94	418	199
GmEF8	2235	1811	2288	3031	2329	1441	1941	1222	1661	1515	1900	741	2360	2332
GmEF9	134	277	142	200	64	118	290	105	418	298	555	185	1651	134
GmEF10	72	115	81	138	117	50	124	38	93	87	132	77	165	174

Table S2. Primers used in the paper.

Gene	Sequences
Soybean actin Forward	ACATTGTTCTTAGTGGTGGCT
Soybean actin Reverse	CTGTTGGAAGGTGCTGAG
GmEF1 Forward	GGTGGCATCCCCTTCTGATG
GmEF1 Reverse	AGCGTACTGTTCCCACAACA
GmEF2 Forward	TGGAACGGGCCTTGCTTATT
GmEF2 Reverse	CCTCACGAACACTGCCAGAT
GmEF3 Forward	GAAGCGTGGTTATGTTGCC
GmEF3 Reverse	CCAATCTGACCAGGGTGG
GmEF4 Forward	TGCCTGGGTGCTCGACAAGCTCAAA
GmEF4 Reverse	GTCCAGGAGCATCAATGACCGTGCA
GmEF5 Forward	GTAAGGAAAAGGTTCACATCAGT
GmEF5 Reverse	CCCAGGCATACTTGAAAGAC
GmEF6 Forward	AGGAGCTCTTCGGAGTGGAT
GmEF6 Reverse	TGTGCCCACCAATATGGGAC
GmEF7 Forward	GTTCCACTGCGAGATCCCAA
GmEF7 Reverse	CCTCACGAACACTGCCAGAT
GmEF8 Forward	GGCTGATTGTGCTGTCCTT
GmEF8 Reverse	GGTAGTGGCATCCATCTTGTTA
GmEF9 Forward	TCAAGCGTGGTTTTGTTGCG
GmEF9 Reverse	ATGTGGGAGGTGTGGCAATC
GmEF10 Forward	ATGAGCCCAAGAGACCCTCA
GmEF10 Reverse	CCATACCGGGCTTCAAGACA
GmEF4-3301 Forward	GGACTCTTGACCATGATGGGTAAGGAAAAGGTTCA
GmEF4-3301 Reverse	ATTCGAGCTGGTCACCCTTCTTCTTGGCGGCAG
GmEF4-GFP Forward	GCCCAGATCAACTAGTATGGGTAAGGAAAAGGTTCA
GmEF4-GFP Reverse	TCGAGACGTCTCTAGACTTCTTCTTGGCGGCAG

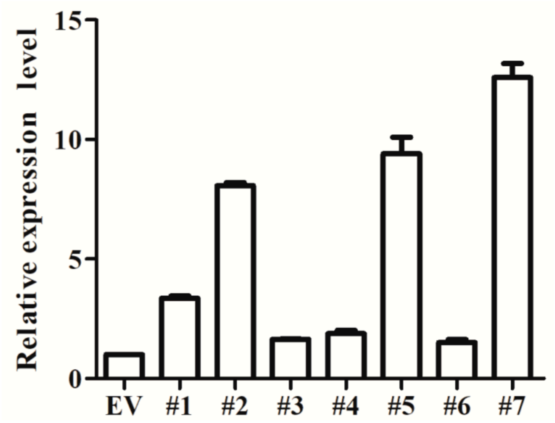

Figure S1. qRT-PCR analyses of GmEF4 expression in GmEF4-OE and EV-control transgenic soybean hairy roots.
